# Premenopausal Obesity and Breast Cancer Growth Rates in a Rodent Model

**DOI:** 10.3390/nu8040214

**Published:** 2016-04-11

**Authors:** Shawna B. Matthews, John N. McGinley, Elizabeth S. Neil, Henry J. Thompson

**Affiliations:** Cancer Prevention Laboratory, Colorado State University, Fort Collins, CO 80523, USA; shawna.matthews@ucdenver.edu (S.B.M.); john.mcginley@colostate.edu (J.N.M.); elizabeth.neil@colostate.edu (E.S.N.)

**Keywords:** apoptosis, breast cancer, cell proliferation, obesity, premenopausal, tumor growth

## Abstract

Obese premenopausal women with breast cancer have poorer prognosis for long term survival, in part because their tumors are larger at the time of diagnosis than are found in normal weight women. Whether larger tumor mass is due to obesity-related barriers to detection or to effects on tumor biology is not known. This study used polygenic models for obesity and breast cancer to deconstruct this question with the objective of determining whether cell autonomous mechanisms contribute to the link between obesity and breast cancer burden. Assessment of the growth rates of 259 chemically induced mammary carcinomas from rats sensitive to dietary induced obesity (DS) and of 143 carcinomas from rats resistant (DR) to dietary induced obesity revealed that tumors in DS rats grew 1.8 times faster than in DR rats. This difference may be attributed to alterations in cell cycle machinery that permit more rapid tumor cell accumulation. DS tumors displayed protein expression patterns consistent with reduced G1/S checkpoint inhibition and a higher threshold of factors required for execution of the apoptotic cell death pathway. These mechanistic insights identify regulatory targets for life style modifications or pharmacological interventions designed to disrupt the linkage between obesity and tumor burden.

## 1. Introduction

There is a longstanding awareness within the public health community of the importance of identifying subpopulations of individuals that respond differently to various environmental exposures including those that relate to energy balance [1]. Along these lines, the reported impact of obesity on the risk for developing breast cancer is dichotomized by menopausal status [2,3]. While obesity has been widely observed to have no effect or to be protective against breast cancer in premenopausal women, excess adiposity is associated with a significant increase in breast cancer risk in postmenopausal women [2,4,5,6]. However, recent work has indicated that the lack of effect of obesity in premenopausal women may not apply to those individuals who are at increased breast cancer risk defined using the Gail score metric [7]. The Gail model was developed in 1989 as a tool to model the influence of risk factors, including current age, age at menarche, parity, and family history, among other factors, on 5-year and lifetime invasive breast cancer risk [8]. Consistent with this observation [7], we recently reported that in a polygenic premenopausal model for obesity and breast cancer, the occurrence of breast cancer is markedly increased in rats that are susceptible to dietary induced obesity *versus* those that are resistant [9].

While more work is required to clarify the reported differences in breast cancer risk by menopausal status, larger breast tumors are generally detected in obese *versus* normal weight women irrespective of their menopausal status, and tumor size is an important prognostic marker [10]. Mechanisms frequently cited as underlying the obesity and breast cancer link include deregulated glucose homeostasis and associated insulin resistance; increased prevalence of obesity induced chronic inflammation and/or cellular oxidation; increased peripheral aromatization of testosterone to estrogen; and deregulated adipokine metabolism; however, we found limited evidence that these mechanisms were involved in the premenopausal rat model [9]. Moreover, it is possible that larger tumors are found in obese women because they are simply more difficult to detect and thus detected at a later point in their development [11]. An alternative hypothesis is that unappreciated cell autonomous effects of excess energy availability, which leads to excess adipose tissue accumulation, alter kinetic aspects of the carcinogenic process. Identification of such effects and the contributing mechanisms might offer new avenues for risk reduction and improved prognosis either through life style modifications or pharmacological interventions.

The experiments reported herein were conducted using tissue obtained from a previously reported study in which mammary cancer incidence, multiplicity, and burden were increased and cancer latency was reduced in dietary obesity sensitive *versus* resistant rats [9]. The rats in that study were young and in the early stages of excess body fat accumulation, thus enhancing the opportunity to study effects on kinetics of tumor growth in the absence of confounding effects due to obesity per se. Both rat strains were fed the same diet with 32% of dietary calories as fat, in contrast with studies performed in mice where obesity is induced by feeding a supra-physiological level of dietary fat (45%–60% of calories), and the lean control mice are fed a low fat diet (14% of calories). Other models have investigated lower fat levels (33%–45% kcal) or genetic models of obesity as reviewed in [12]. Secondly, the majority of the breast carcinomas induced were sex steroid hormone positive, a molecular subtype of breast cancer that obesity is considered to promote in women [13,14,15]. In comparison, the majority of breast cancers arising from mouse mammary gland are sex steroid hormone negative [16].

In this study, we tested the hypothesis that breast cancer growth rates are faster in dietary obesity sensitive (DS) *versus* dietary obesity resistant (DR) tumors, a question that has not previously been addressed. Finding evidence consistent with this hypothesis, tumors were interrogated to identify the cellular processes and molecular mechanisms that accounted for this effect.

## 2. Materials and Methods

### 2.1. Study Design

Design and implementation of the carcinogenesis experiment has been previously reported [9]. Briefly, breeder pairs (approximately 30 pairs each Levin dietary obesity resistant (DR) and dietary obesity sensitive (DS)) were obtained from Taconic (Taconic, Hudson, NY, USA) at 5–7 weeks of age. In-house breeding was conducted using a Poiley rotational breeding scheme, in which breeder pairs are systematically rotated in each breeding cycle [17]. Pups were weaned at 3 weeks of age and were immediately switched to the same purified diet. Post-weaning, rats were housed 3 per cage, maintained on 12 h light:dark cycle at 24 ± 2 °C with 30% relative humidity, and given ad libitum access to purified diet and distilled water. Animals were weighed weekly. To initiate mammary carcinogenesis according to the rapid emergence model first developed by our laboratory [18], female DR (*n =* 103) and DS (*n =* 101) rats were injected intraperitoneally (50 mg/kg) with 1-methyl-1-nitrosourea (MNU) (Ash Stevens, Detroit, MI- prepared fresh in acidified saline) at 21 days of age as previously described [19]. Bi-weekly palpations for detection of mammary tumors began 24 days post-carcinogen and continued until study termination. The study was terminated 63 days post-carcinogen when rats were 84 days of age. Rats were skinned and mammary gland chains were examined under translucent light; grossly visible tumors were excised, weighed, and processed for histopathological analysis as previously described [20]. All animal studies were performed in accordance with the Colorado State University Institutional Animal Care and Use Committee.

### 2.2. Immunohistochemical Evaluation of Proliferation (Ki67)

Four micron sections of formalin fixed paraffin embedded (FFPE) tumors were assessed for Ki67 expression using immunohistochemistry methods as previously described [21]. Briefly, a Ki67 rabbit monoclonal primary antibody, clone SP6 (Thermo Scientific, Waltham, MA, USA) was diluted 1:200 in phosphate buffered saline + 0.05% tween-20 (PBS-T) + 10% NDS and incubated for 60 min, 3 × 5 min washes in PBS-T, followed by incubation with a biotinylated donkey anti-rabbit secondary antibody (Jackson ImmunoResearch, West Grove, PA, USA); diluted 1:1000 in PBS-T + 10% NDS and applied for 30 min, 3 × 5 min rinses in PBS-T followed by Stable DAB (Invitrogen, Grand Island, NY, USA) for 10 min and counterstained with dilute hematoxylin (1:10) for 3 min. Rat colon tissue was included as a positive control for Ki67 staining. In addition, both tumor size and heterogeneity were used to ascertain the number and location of digital image fields captured at 400× magnification for analysis based on a method adapted from Regan *et al*. [22]. Briefly, the total number of fields measured was based on the long axis measurement of the tumor section separated into categories: <10 mm, ≥10 mm and <15 mm, ≥15 mm and <20 mm, >20 mm; the number of microscopic image fields captured in each category were 5, 10, 15 and 20, respectively. The section was then scanned visually by the observer at low power to determine the approximate percent area of intensely stained regions within the tumor and fields captured accordingly. For example, a 12 mm tumor with 30% intensely stained area or “hot spots” would have 3 fields captured from the “hot spot” area(s) and the remaining 7 captured from typical fields. Percentages resulting in field fractions were rounded up to the next field. Expression was analyzed using the ImmunoRatio plugin for ImageJ open source image analysis software [23].

### 2.3. Histological Evaluation of Apoptosis

Digital images from 4 µm hematoxylin & eosin (H & E) stained sections of FFPE tumors were captured at 400× magnification. The number and location of H & E fields captured corresponded to those from Ki67 stained serial sections*.* A macro was written in Image Pro Plus v4.5 (Media Cybernetics, Inc., Rockville, MD, USA) to facilitate manual tagging analysis of each image. Analysts were blinded as to tumor identity and each field was tagged by one researcher then checked by a different researcher to ensure that all cells were tagged and to align agreement upon tags. Necrotic cells were not tagged. Apoptotic and mitotic indices were determined as number of tagged per total cells in a high-powered field, generally 700 to 1000 cells per field, using a census counting technique.

### 2.4. Lysate Preparation

DR and DS tumor lysate for analysis of protein expression was prepared using Tissue Protein Extraction Reagent (T-PER) from Pierce (Rockford, IL, USA), with all buffers containing 1× HALT protease and phosphatase inhibitor cocktail (Pierce, Rockford, IL, USA). Briefly, ~200 mg flash-frozen tumor tissue was pulverized using mortar and pestle, then 2 mL T-PER was added and incubated with the lysate on ice for 20 min. Lysate was centrifuged at 12,000× *g* for 20 min and the clear supernatant containing soluble proteins was transferred to a separate tube and aliquoted; the pellet containing nuclei, membranes, and insoluble material was discarded. Protein concentration was determined by Bradford assay and samples were diluted to equal concentration of protein per mL in ice-cold T-PER buffer containing 1× HALT protease/phosphatase inhibitor.

### 2.5. Western Blot-Based Detection/CE-Based Protein Expression

Tumor lysate from the high mitotic index subset was evaluated using sodium dodecyl sulfate-polyacrylamide gel electrophoresis (SDS-PAGE) under denaturing and reducing conditions with detection. Briefly, lysates were prepared to contain final concentration 1× Nu-PAGE LDS sample buffer (Life Technologies, Grand Island, NY, USA) containing a denaturing agent and 0.1 M DTT as a reducing agent. Samples were boiled at 95 °C for 5 min, then 60 µg protein from each sample was loaded into a 4%–12% Tris-Glycine gel (Life Technologies, Grand Island, NY, USA) and run at 125 V for 90 min to separate proteins. Following SDS-PAGE, samples were transferred from the gel to a polyvinylidene fluoride (PVDF) membrane by applying 25 V for 2 h at 4 °C. Quality of transfer was evaluated by staining the gel and membrane with Coomassie and Ponceau S stains, respectively (Bio-Rad, Hercules, CA, USA). Membranes were blocked with 5% nonfat dry milk in Tris-buffered saline + 0.1% Tween-20 (TBS-T), and were incubated overnight at 4 °C with primary antibodies diluted in 5% bovine serum albumin (BSA) in TBS-T. For detection, membranes were washed 3× with TBS-T, incubated with appropriate secondary antibodies directed against the host species of the primary antibody, then washed 3× with TBS-T. Membranes were incubated with Clarity Enhanced Chemiluminescence Reagents (ECL) (Bio-Rad, Hercules, CA, USA) and images were collected within the linear range of detection (below pixel saturation) using a ChemiDoc imager (Bio-Rad, Hercules, CA, USA) or a WES capillary electrophoresis system (Protein Simple, Santa Barbara, CA, USA). Signal specificity was confirmed by comparing observed bands to the Amersham Full-Range Rainbow pre-stained protein ladder (GE Healthcare, Lafayette, CO, USA).

### 2.6. Immunoprecipitation

Immunoprecipitation was performed using the Protein G Dynabeads kit from Novex (Thermo Fisher, Grand Island, NY, USA) according to the manufacturer’s protocol. Briefly, Dynabeads were resuspended and 50 µL was transferred to a tube then placed on the magnet to separate beads from supernatant. Ten µg mouse anti-rat E2F1 antibody (Santa Cruz, Dallas, TX, USA) was incubated with rotation for 10 min at room temperature. Tube was placed back on the magnet and unbound antibody and supernatant was removed. Beads were washed 1× with PBS-T wash buffer, tube was placed on the magnet, and wash supernatant was removed. Five hundred µL each sample (1 mg/mL) was added to the beads with rotation for 10 min at room temperature. The antibody-antigen-Dynabeads complex was washed 3× with wash buffer. After each wash, beads were placed back on the magnet, supernatant was removed, and beads were resuspended in fresh wash buffer. Immediately prior to running the samples, 21 µL elution buffer and 7 µL 4× LDS sample buffer (Life Technologies, Grand Island, NY, USA) and 0.1 M DTT were added, mixed, and tubes were heated for 10 min at 70 °C. Tubes were put on magnet to remove beads and 10 µL supernatant was run via SDS-PAGE as described in section B.8. Blocking and antibody incubation steps were performed as described in section B.8; to prevent detection of antibody heavy and light chains, TrueBlot secondary antibodies were used (detects only native/non-reduced/denatured antibodies (Rockland, Limerick, PA, USA) and detected via chemiluminescence.

### 2.7. Statistical Methods

#### 2.7.1. Apoptotic/Mitotic Indices

To determine the probability a cell undergoing apoptosis or mitosis, probabilities for each of these phenomenon detected via histological means were calculated. While Poisson distributions are commonly used to model tumor data, count data from H & E stained fields was demonstrated to be overdispersed compared to a reference Poisson model. One possible reason for overdispersion, aside from incorrect model usage, is positive correlation among observations [24]. Given the overdispersion of the count data, probabilities of apoptosis and mitosis were determined using a negative binomial distribution, which arises from mixing a Poisson process with a gamma distribution for the Poisson parameter and hence is overdispersed compared to a reference Poisson model.

#### 2.7.2. Western Blot/CE-Based Evaluation of Protein Expression

For Western blot, to quantify signal, densitometry was performed after correcting for background using the rolling disk method, size 20. Signals are expressed as density/mm^2^ normalized to glyceraldehyde 3-phosphate dehydrogenase (GAPDH) as a loading control. For capillary electrophoresis, chemiluminescent signal was taken as peak area and normalized to GAPDH as a loading control.

#### 2.7.3. Multivariate Statistical Modeling

Principal components analysis (PCA) is a method to analyze large multivariate dataset which summarizes a set of correlated variables by transforming them, by means of an Eigen decomposition, into a new set of uncorrelated variables, reducing the dimensionality of the original high dimensional dataset [25,26,27,28]. The first principal component (PC) is the linear combination of the features that passes through the centroid of the full dataset while minimizing the square of the perpendicular distance of each point to that line; each subsequent PC is constructed in a similar manner while being mutually orthogonal [25].

## 3. Results

### 3.1. Chemically-Induced Mammary Carcinogenesis Is Accelerated in DS Rats

**Figure 1:**
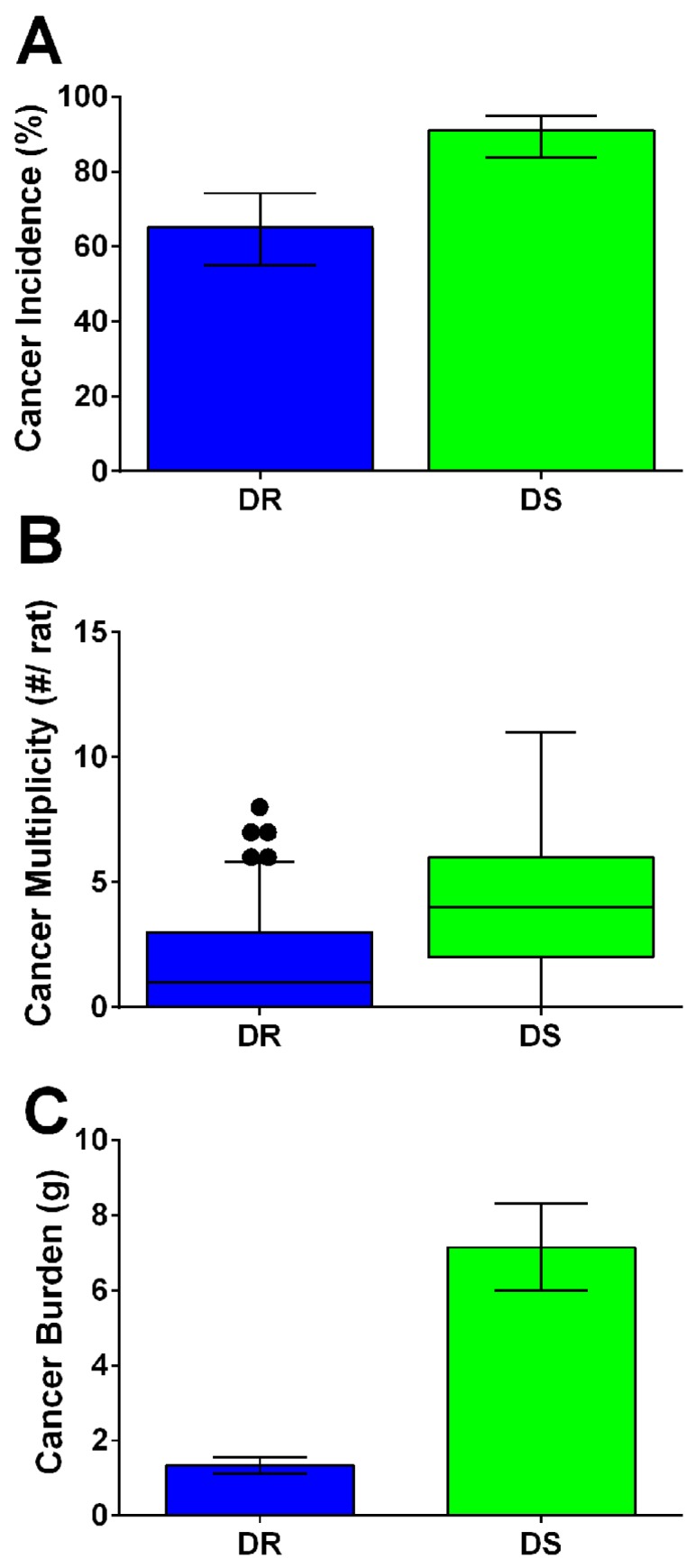
Obesity accelerates mammary carcinogenesis in dietary obesity sensitive (DS) rats. Mammary carcinogenesis was initiated by injecting rats intraperitoneally with 50 mg/kg 1-methyl-1-nitrosourea (MNU) at 21 days of age. Study was terminated 63 days (9 weeks) after carcinogen; only palpable confirmed mammary adenocarcinomas were included for analysis. In Figure 1A–C, groups with different letters significantly differ. (**A**) Cancer incidence, percentages (95% CI); (**B**), cancer multiplicity, means (95% CI); (**C**), cancer burden, means (95% CI). Values were higher in DS compared to dietary obesity resistant (DR) rats.

Mammary carcinogenesis was induced by injecting rats with the carcinogen MNU (50 mg/kg) at 21 days of age. Tumors were harvested at necropsy 63 days post-carcinogen. Following diagnosis, palpable histopathologically confirmed mammary adenocarcinomas were compared between DR and DS rats. DS rats displayed higher cancer incidence (91%) than observed in DR rats (65%) (Figure 1A). DS rats displayed higher cancer multiplicity (total cancers per rat) and cancer burden (sum tumor weight per rat) compared to DR (Figure 1B,C; *p* < 0.001 for all analyses).

### 3.2. Tumor Burden, Body Weight, and Adiposity

DS rats had an average tumor burden per rat (g/rat) that was 5.6 times greater than observed in DR rats [9]. This marked difference in tumor burden occurred when the rats had only been on study for 63 days. At that time, the average body mass of DS rats was 15% greater, visceral fat stores normalized to tibia length on average were 2.7 times larger, and the size of adipocytes in the mammary gland was 38% greater in DS *versus* DR (Table 1). The fact that the 5.6 fold difference in tumor burden was observed when differences in adiposity were relatively small provided the rationale for investigating cell autonomous processes related to tumor burden.

### 3.3. Change in Tumor Mass

In the rat model, mammary tumors are detected by physical palpation of the rat twice per week; tumors are generally detected when they have a mass of 100 mg. In this study, rats were euthanized at the same number of days from carcinogen administration and the mass of each tumor was determined at necropsy. These data (date first detected by palpation and tumor mass at necropsy) were obtained for 143 histologically confirmed mammary carcinomas in the DR group and 259 mammary carcinomas in the DS group.

Tissue size is normally strictly controlled to maintain a constant cell number: one cell replicates to replace one cell that has died. This is quantified in Equation (1), which models the size of a tissue as a balance between proliferation and death [29].

Δ*S* = *n* (*k_P_* − *k_D_*) = 0 (1)

**Figure 2:**
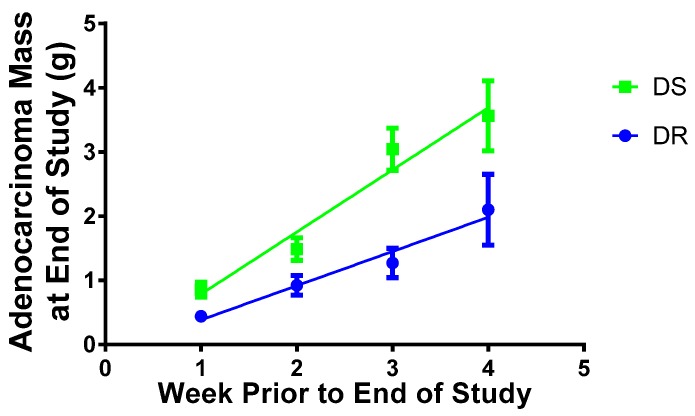
Estimation of tumor growth rates in dietary obesity sensitive (DS) and dietary obesity resistant (DR) rats. Week Prior to End of Study: a value of 0 is the end of study; week 4 indicates the tumor was detected 4 weeks prior to the end of study and had a longer timeframe during which to grow than tumors detected 3, 2, or 1 week prior to the end of study. Values are means ± SEM, *n* = 259 DS and 143 DR mammary carcinomas. The regression coefficients for each line (slope): DR 0.533 ± 0.070 g/week; DS 0.968 ± 0.140 g/week, *p* < 0.01.

In this equation, Δ*S* is the change in tissue size, *n* represents cell number, *k_P_* is the rate of cell proliferation, and *k_D_* is the rate of cell death. When Δ*S* = 0, the rate of proliferation is equivalent to the rate of death and the tissue is in homeostasis, *i.e.*, does not change in size. The occurrence of a tumor represents a failure of tissue size homeostasis. As both DR and DS rats developed tumors, each tumor had a positive value for Δ*S* in Equation (1). Thus, we set out to determine how Δ*S* (tissue size) changed over time in tumors from DR and DS rats. If tumors were accumulating cell mass at the same rate in DS and DR rats, tumors palpated on the same day would be expected to have the same mass at study termination (DR Δ*S* = DS Δ*S*). However, the alterative hypothesis proposes that DS tumors accumulated cells at a faster rate than DR tumors (DR Δ*S* ≠ DS Δ*S*). By taking the data for the different time points at which tumors were detected by palpation and regressing M_T_, mass of the tumor determined at necropsy, on week at which it was first detected by palpation, the change in Δ*S* over time was computed for DR and DS tumors via regression analysis (Figure 2). The slope of the regression line for DS tumors is nearly double that of DR tumors (slope: DR 0.533 ± 0.070 g/week; DS 0.968 ± 0.140 g/week; a 1.82-fold increase in DS over DR, *p* < 0.01). This suggests a greater imbalance in the *k_P_* and *k_D_* terms of Equation (1) for DS tumors, a finding that suggests that tumors grow faster in DS rats.

### 3.4. Cellular Processes Associated with Tumor Growth

**Figure 3:**
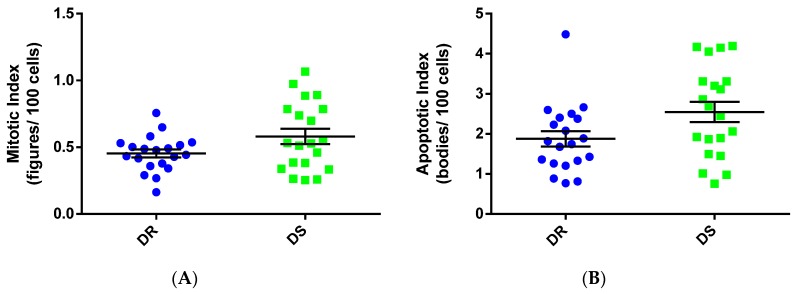
Mitotic and apoptotic indices in mammary carcinomas from dietary obesity sensitive (DS) and resistant (DR) rats. Bars indicate means ± SEM.

A subset of tumors used for the analysis shown in Figure 2 was selected for subsequent cellular and molecular evaluation. Twenty matched pairs of tumors were chosen based on size and palpation date to emulate the growth characteristics shown in Figure 2 (*n* = 20 each DR and DS) in an effort to detect mechanisms that could account for the faster growth rate of DS tumors. The first step in the investigation established that there was no difference in average cell number per field, and by extension no difference in cell size between DR and DS tumors (DR, 838.7 ± 19.1; DS, 863.2 ± 15.3 cells per field, mean ± SEM), making it unlikely that the differences in Δ*S* were due to cell hypertrophy. Therefore, we proceeded to interrogate the processes of cell proliferation and apoptosis. Using high-powered fields of H & E stained tumor sections, mitotic and apoptotic indices (expressed as number of mitotic figures/total cells per field and number of apoptotic bodies/total cells per field, respectively, and the mean calculated for all fields analyzed per tumor) were determined. Under a negative binomial distribution, probability of a given cell undergoing mitosis was higher in DS tumors than in DR tumors (DR, 0.448 ± 0.131; DS 0.571 ± 0.252 mitotic figures per field; *p =* 0.063, Figure 3A). Similarly, the probability of a given cell from DS tumors undergoing apoptosis was higher than in DR tumors (DR 1.877 ± 0.829; DS 2.616 ± 1.173 apoptotic cells per field; *p* = 0.028, Figure 3B).

### 3.5. Cell Cycle Length and Duration of Apoptosis

#### 3.5.1. Cell Cycle Length

The protein Ki67 was identified in 1991 by Gerdes *et al*. as a nuclear protein expressed in proliferating cells in all phases of the cell cycle [30]. Analysis of immunoreactive nuclear area revealed no difference in proliferating fraction of cells in the 20 pairs of DS *vs*. DR tumors (DR, 9.0% ± 0.7; DS, 9.4% ± 0.9, mean ± SEM). In the average cell, cell cycle duration is estimated at 24 h, with mitosis lasting approximately 1 h [31]. Ki67 % nuclear immunoreactivity represents the proliferating fraction of cells. In both DR and DS tumors, the majority of cells were negative for expression of nuclear Ki67, indicating that the proliferative fraction of these tumors corresponds to a small percentage of cells. To determine the relationship between mitotic index and proliferative fraction within tumors, we estimated cell cycle duration as shown in Equation (2).

(2) $$\text{Cell cycle duration }\left(h\right)=\frac{\text{Proliferative fraction }\left(\%\right)}{\text{Mitotic index }\left(\%\right)}$$

Cell cycle duration in DS tumor cells was approximately 16.5% shorter than in DR tumor cells (DR, 21.2 ± 1.7 h; DS 17.7 ± 1.3 h, mean ± SEM). These data are consistent with the hypothesis that DS tumor cells progress through the cell cycle at an accelerated rate compared to DR, *i.e.*, the proliferating fraction of cells within DS tumors produced more daughter cells per unit time than occurred in DR tumors.

#### 3.5.2. Apoptotic Duration

Rates of proliferation (*k_p_*) and death (*k_D_*) can be split into terms of cell number (proliferative number = *P*_n_ (Ki67% immunoreactivity); dead number = *D*_n_ (apoptotic index)) and process duration (proliferation duration = *P*_d_ (estimated cell cycle duration); death duration = *D*_d_). Change in tissue size (Δ*S*) values are equal to final tumor mass divided by h elapsed between palpation and study termination. As an estimate for *n*, slopes from Figure 2 were converted from g/week to cells gained/h accounting for the mass of a single cell (1 × 10^−^^9^ g) [32,33]. Using available data, we solved for *D*_d_, apoptotic duration, using Equation (3).

(3) $$D_{d}=\frac{D_{n}}{\left(\left(\frac{P_{n}}{P_{d}}\right)-\left(\frac{\Delta S}{n}\right)\right)}$$

Solving for *D*_d_ of each tumor, apoptotic duration was estimated to be 25.5% longer for a cells within DS *versus* DR; *i.e.*, the total number of cells eliminated by apoptosis per unit of time would be lower in DS than in DR tumors. However, the difference in *D*_d_ between DR and DS tumors did not reach statistical significance (DR 4.7 ± 0.6 h; DS 5.9 ± 1.0 h, mean ± SEM).

#### 3.5.3. Multivariate Analyses of Cell Proliferation and Apoptosis Data

The data for cell proliferation (mitotic index and cell cycle duration) and apoptosis (apoptotic index and duration of apoptosis) were subjected to multivariate regression analysis to determine the extent to which they explained differences in tumor cell mass. The regression model explained 55.4% of the variation in tumor mass (*r*^2^ = 0.554, *p* < 0.001). Unsupervised principal components analysis of the data was performed and a 4 component model was fitted with an R^2^X (cum) = 0.98 and Q2 (cum) = 0.66. These data were subjected to multivariate analysis which discriminated DS from DR tumors (Hotelling statistic, *p* = 0.025) providing additional support for the distinction between DR and DS tumors based on these measurements of proliferation and apoptosis.

### 3.6. Effects on Cellular Machinery

In order to have greater sensitivity to determine what factors were driving the faster growth rates in DS tumors, we focused our analyses on a subset of 10 DS and 10 DR tumors with the highest mitotic indices of the tumors evaluated in Section 3.4.

#### 3.6.1. Cell Cycle

**Figure 4:**
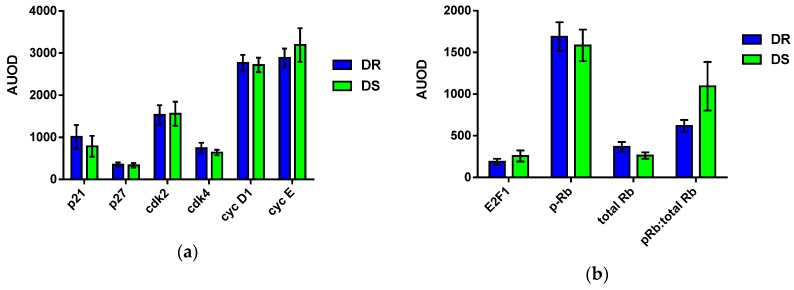
Expression levels (AUOD, arbitrary units of optical density normalized to glyceraldehyde 3-phosphate dehydrogenase (GAPDH) for proteins involved in G1/S transition. Tumors from dietary obesity sensitive (DS) and dietary obesity resistant (DR) rats were evaluated. Values are means ± SEM. Because expression of these proteins is interrelated, the overall impact of obesity status was evaluated by multivariate analysis techniques (Section 3.6.3).

Most variability in cell cycle duration is due to the rate of transit of the G1/S restriction checkpoint [31,34]. Upon hyperphosphorylation of the retinoblastoma (Rb) protein at serines 807 and 811 by cyclin dependent kinases (cdks) 2 and 4, pRb changes conformation and dissociates from the E2F1 transcription factor, which is then free to stimulate transcription of factors required for DNA synthesis in S phase [31]. Given the shorter estimated cell cycle duration observed in DS tumor, we evaluated expression of proteins which regulate the passage from G1 into S phase. Expression levels of the majority of G1/S restriction checkpoint proteins, including p27, cdk2, cdk4, and cyclin D1 did not differ (Figure 4). In DS tumors compared to DR tumors, a trend towards reduced expression of the cyclin dependent kinase inhibitor p21, with concomitant trends towards increased expression of pro-proliferation proteins cyclin E and E2F1, was observed. Tumors from DS rats display a significantly higher ratio of phosphorylated Rb to total Rb compared to DR rats. In support of the hyperphosphorylated state of Rb, immunoprecipitation of lysate with antibodies against E2F1 revealed a trend towards reduced Rb in the complex (Supplemental Figure S1). In the scenario of hyperphosphorylation of Rb, E2F1 is free to exert transcriptional control over a number of proteins, including the S phase protein cdc6, whose promoter contains an E2F-binding site [35]. Expression of cdc6 was increased in DS tumors compared to DR tumors (Supplemental Figure S1). These data suggest that proliferating cells in DS tumors have fewer obstacles at the transition from G1 to S phase and so may demonstrate faster cell cycle transit.

#### 3.6.2. Effects on Apoptotic Machinery

**Figure 5:**
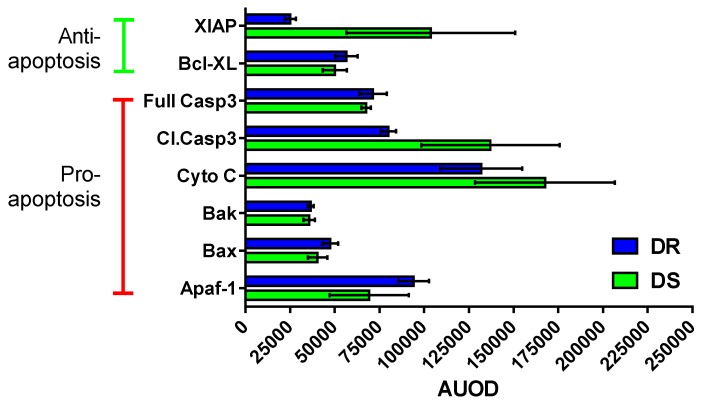
Expression levels (AUOD, arbitrary units of optical density normalized to GAPDH) for proteins involved in apoptosis. Tumors from dietary obesity sensitive (DS) and dietary obesity resistant (DR) rats were evaluated. Values are means ± SEM. Because expression of these proteins is interrelated, the overall impact of obesity status was evaluated by multivariate analysis techniques (Section 3.6.3).

To evaluate what aspect of the apoptotic process was being impacted in DS *versus* DR tumors, expression of proteins involved in apoptosis was undertaken. In many cases DR and DS tumors displayed similar expression levels of both pro- and anti-apoptotic proteins (Figure 5); however, the level of XIAP was elevated in DS tumors. DS tumors displayed a trend towards higher cleaved caspase 3 (executioner caspase) and higher cytochrome c expression levels. These data are consistent with the hypothesis that higher levels of pro-apoptotic stimuli may be required to induce apoptosis in DS tumors which would cause the persistence of the morphological features of apoptosis, and thus an apparent higher apoptotic rate (Figure 3), but actually reduce the number of cells eliminated per unit of time in DS tumors.

#### 3.6.3. Multivariate Analysis of Tumor Growth Characteristics

All of the growth characteristics of DR and DS tumors, while presented individually, are interrelated at a cellular level as indicated by Equations (1)–(3). In order to simultaneously evaluate the relationship between characteristics of proliferation and death on tumor mass, all variables were imported into the Simca-P+ multivariate data analysis program (Umetrics, San Jose, CA, USA). Partial least squares projections to latent structures (PLS) analysis is a method used to visualize the effect of interrelated X predictor variables on Y response variables. In the current study, *n* = 29 X variables (including count data, immunohistochemistry data, and protein expression data) and 1 Y variable (tumor mass) were evaluated by PLS analysis. Coefficients of the 29 X variables represent the strength of their predictive effect on Y. Specifically, whereas large negative values have strong inverse associations with Y, large positive values have strong positive associations with Y. Coefficients for the interaction of each X variable in the first (only) component is shown for tumor mass (Supplemental Figure S2).

The three X variables with strongest positive correlation with tumor mass (*i.e.*, increased in larger tumors) were cleaved caspase 3, cytochrome c, and anti-apoptotic protein X-linked inhibitor of apoptosis (XIAP). XIAP has been found to bind to cleaved caspase fragments and interfere with downstream induction of the morphological changes associated with apoptosis. This co-association of XIAP and cleaved caspase 3 with larger tumor mass suggests that XIAP may interfere with caspase activity in larger tumors. Cdc6 and immunoprecipitated E2F1 were also positively correlated with tumor mass. However, the magnitude of this correlation was much smaller than that observed with apoptotic proteins. Conversely, the three X variables with strongest inverse correlation with tumor mass (*i.e.*, lower in larger tumors) were pro-apoptotic proteins Apaf-1, total caspase 3, and Bax. Cdk2 and cyclin E were inversely correlated with tumor mass, as was immunoprecipitated Rb. This suggests that whereas expression levels of cdk2 and cyclin E do not increase with tumor mass, activity of this cdk/cyclin complex, resulting in phosphorylation and inactivation of Rb, may increase in larger tumors.

## 4. Discussion

Obese women with breast cancer are more likely to have larger tumors compared to their lean counterparts. Limited attention has been directed to this situation despite the fact that it is a harbinger of poor prognosis. Due to the magnitude of the effect on tumor mass that was observed in tumor bearing DS *versus* DR rats, we decided to deconstruct the mechanism behind increased tumor size in a premenopausal model for obesity and breast cancer. As shown in Figure 2, strong evidence was obtained that tumor mass accumulation was approximately twice as rapid in DS *versus* DR rats over the identical period of time, an effect that could not be accounted for by differences in tumor cell size. Given the very short period of time over which both obesity and carcinogenesis occurred [9], we decided to focus on cell autonomous mechanisms including the balance between cell proliferation and apoptotic death to investigate the factors underlying the faster growth rates of DS tumors, a question that to our knowledge has not previously been investigated with a focus on tumor growth kinetics.

Using the computations detailed in Equations (1)–(3), the processes resulting in the more rapid accumulation of cell mass in tumors from DS rats appear to indicate alterations in the checkpoint barriers to cells moving from G1 to S (Figure 2), rather than to differences in the number of cells in the proliferative pool. The evidence for this was of two types. First, solving Equation (2) revealed a 16.5% decrease in cell cycle duration in DS *versus* DR tumors, and solving Equation (3) indicated a trend towards a 25.5% increase in the estimated duration of the apoptotic process in DS *versus* DR tumors. Such differences, while small individually, clearly could account for more rapid cell accumulation in DS tumors of the magnitude observed when operating together. This assessment is supported by evidence from other laboratories that the accumulation of a driver gene mutation conferring selective growth advantage and resulting in clinically detectable disease is attributed to an imbalance between cell proliferation and death of 0.04%, or only 4 in 10,000 cells [36,37]. Second, in investigating the cellular machinery responsible for proliferation and apoptosis, evidence was found that was consistent with the effects on the cell cycle being mediated by hyperphoshorylation of Rb in DS tumors and that the effect on apoptosis was mediated, at least in part, by interference with the activity of executioner caspases via inhibitor of apoptosis proteins such as XIAP (Figure 3).

The question that emerges from this analysis is “what accounts for these effects?” Given that the initiation of breast cancer with MNU is sufficient for tumor induction and that the timeframe of this experiment was 63 days following carcinogen injection, we judge that it is unlikely that the imbalance between cell proliferation and cell death was due to the occurrence of additional driver gene mutations in tumors in DS rats. Similarly, since plasma indicators of glucose homeostasis, chronic inflammation, and sex steroid metabolism were not markedly different in DS *versus* DR rats at necropsy (63 days after study initiation) [9], a compelling case does not exist to support their involvement. Thus, as an alternative, we call attention to the fact that inhibitor of apoptosis proteins such as XIAP have been reported to be induced by metabolic stress and that it would be expected that a stress response would occur in tissues responding to excess energy intake before systemic effects are observed [38]. With this in mind, the data in Table 1 is informative in showing that adipocytes in the mammary gland were 37% larger in DS *versus* DR rats at the time of necropsy. Whether such a response triggers metabolic stress in adjacent structures, *i.e.*, mammary epithelial cells in premalignant and malignant pathologies, to our knowledge has never been investigated, but merits consideration.

How is increased tumor growth rate linked to the excess supply of nutrients and energy in the development of obesity? Mediators of energy status (energy messengers) communicate with intracellular energy/nutrient sensors that are linked to cell proliferation and growth, cell survival, and cell motility, and endothelial homeostasis. There are at least four intracellular sensing networks impacted by excess energy exposure and among which there is considerable crosstalk: AMPK-mTOR-AKT [39,40,41], sirtuins [40,42,43], peroxisome proliferator activated receptors (PPARs) [44,45], and soluble guanylyl cyclase [46,47,48]. Arguably, the AMPK-mTOR-AKT network, which is comprised of more than 100 nodes, is one of the most commonly deregulated in cancer [49]. Currently available human data as well as data from preclinical models are consistent with the effects of obesity on tumor growth rate being mediated at least in part through one or more of these energy sensing networks. Relative to breaking the link between obesity and tumor burden, restoring regulation of the balance between cell proliferation and apoptosis in cancer-initiated clones of cells undergoing expansion would be anticipated to have favorable effects on clinical outcomes [36,37]. The cellular machinery that accounts for regulation includes proteins involved in the G1/S cell cycle transition and apoptotic induction machinery, including those identified in this study.

## 5. Conclusions

The experiments reported herein provide several pieces of evidence indicating that DS tumors have a faster tumor growth rate than DR tumors, a phenomenon potentially explained by reduced cell cycle duration and a prolonged duration of apoptosis, though more data is needed to confirm these findings. Consistent with these trends, DS tumors displayed hyperphosphorylation of the Rb protein, reduced interaction of Rb with E2F1, loss of repression of E2F1 transcriptional activity, and elevated levels of XIAP, which slows the rate at which apoptotic death is executed. Since multiple regulatory nodes within each pathway are impacted by lifestyle interventions and pharmaceutical agents, the opportunity may exist to intervene in populations of women at risk for breast cancer who are unsuccessful in regulating their body weight. The goal would be to improve prognosis for long term survival by slowing tumor growth rate and the size of tumors detected at the time of initial diagnosis or disease recurrence.

## Figures and Tables

**Table 1:** Visceral and peripheral assessment of adiposity.

Rat Strain ^1^	Perirenal ^2^ (mg/mm)	Retroperitoneal (mg/mm)	Parametrial (mg/mm)	Mammary Gland ^3^ Adipocytes (µm^2^/Field)
DR	6 ± 1	23 ± 4	37 ± 5	1128 ± 44
DS	26 ± 3	65 ± 8	192 ± 37	1561 ± 51

^1^ Dietary obesity resistant (DR); dietary obesity sensitive (DS). Values are means ± SEM. For all parameters DR was significantly different than DS, (*p* < 0.001); ^2^ Units are mg mass divided by length of tibia in mm. ^3^ Units are µm^2^ per 100× field.

## Supplementary Materials

The following are available online at http://www.mdpi.com/2072-6643/8/4/214/s1, Figure S1: Effects on cell cycle machinery, Figure S2: Results of OPLS analysis.

## Abbreviations

The following abbreviations are used in this manuscript:

AUOD: arbitrary units of opical density
DR: dietary obesity resistant
DS: dietary obesity sensitive
FFPE: formalin fixed paraffin embedded
MNU: 1-methyl-1-nitrosourea
APAF: apoptotic protease activating factor 1
BAX: BCL2-Associated X
BCL2: B-cell lymphoma 2
Cdc6: cell division cycle 6
Cdk2: cyclin-dependent kinase 2
Cdk4: cyclin-dependent kinase 4
DAB: 3,3′-diaminobenzidine
E2F1 transcription factor: Retinoblastoma-Associated Protein1
GAPDH: glyceraldehyde 3-phosphate dehydrogenase
H & E: hematoxylin and eosin
IRS: insulin receptor substrate
Ki67: nuclear protein that is associated with and may be necessary for cellular proliferation
P21: cyclin-dependent kinase inhibitor 1
P27: cyclin-dependent kinase inhibitor 1B
PBS-T: phosphate buffered saline + 0.05% tween 20
XIAP: X-linked inhibitor of apoptosis
