# Supplementary material for: Premenopausal Obesity and Breast Cancer Growth Rates in a Rodent Model
Source: Nutrients. 2016 Apr 11;8(4):214. doi: 10.3390/nu8040214 (PMC4848683; doi:10.3390/nu8040214)
Supplement: Supplementary file 1 [file nutrients-08-00214-s001.docx]

Supplementary Materials: Premenopausal Obesity and Breast Cancer Growth Rates in a Rodent Model

Shawna B. Matthews, John N. McGinley, Elizabeth S. Neil and Henry J. Thompson


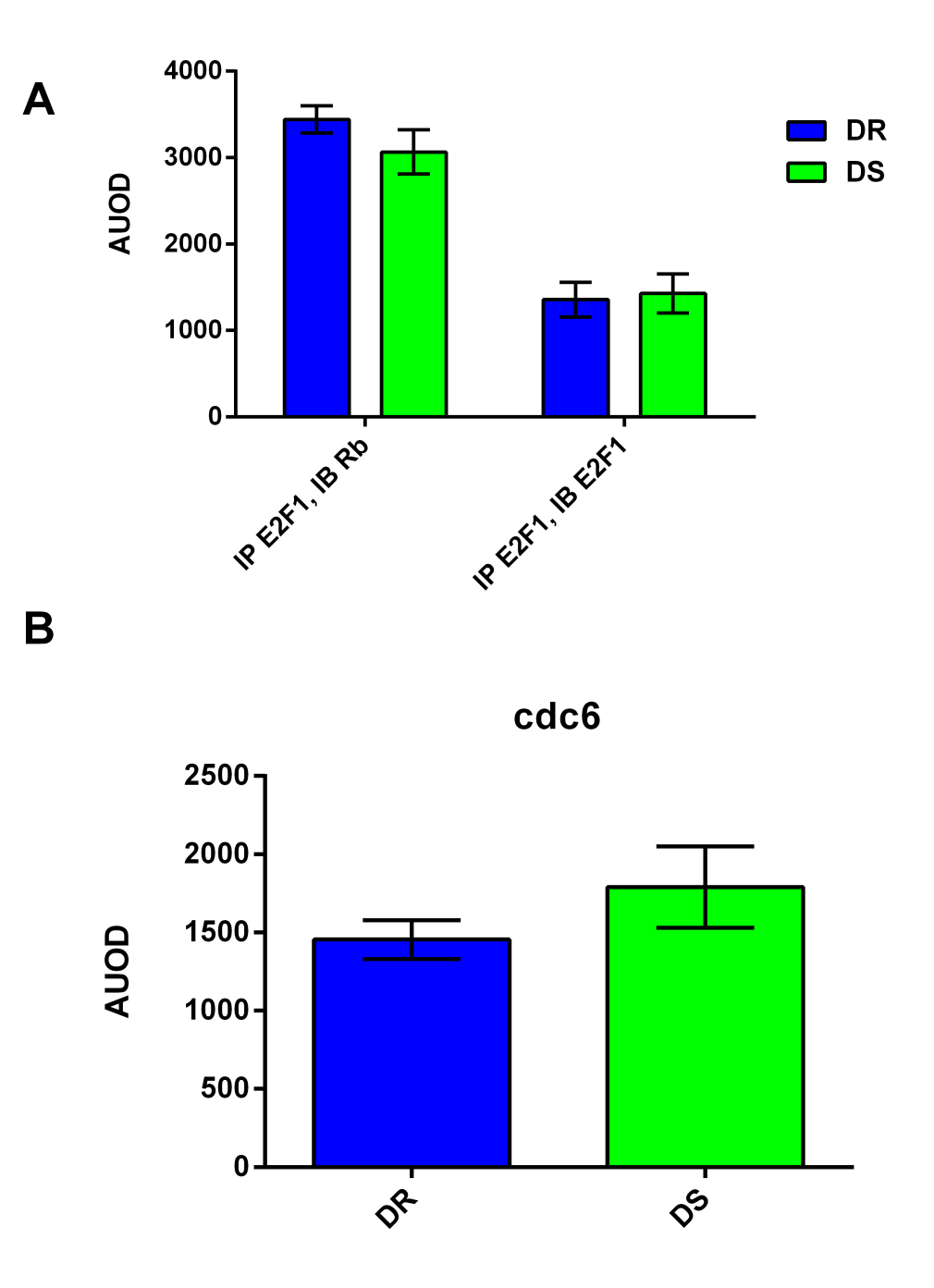


**Figure S1.** Effect of Obesity Sensitivity Status on Cell Cycle Machinery. (**A**) DS rats display hyperphosphorylation of the Rb protein. Lysate from *n* = 9 each high mitotic subset DR and DS tumors were evaluated for expression of G1/S restriction checkpoint proteins. Lysate was immunoprecipitated with E2F1 then assessed for expression of Rb or E2F1. Values are mean density ÷ mm^2^ ± SEM, normalized to GAPDH expression; (**B**) DS rats display increased expression of E2F1 target protein cdc6. Lysate from *n* = 9 each high mitotic subset DR and DS tumors were evaluated for expression of cdc6 via Western blot. Values are mean density ÷ mm^2^ ± SEM, normalized to GAPDH expression.


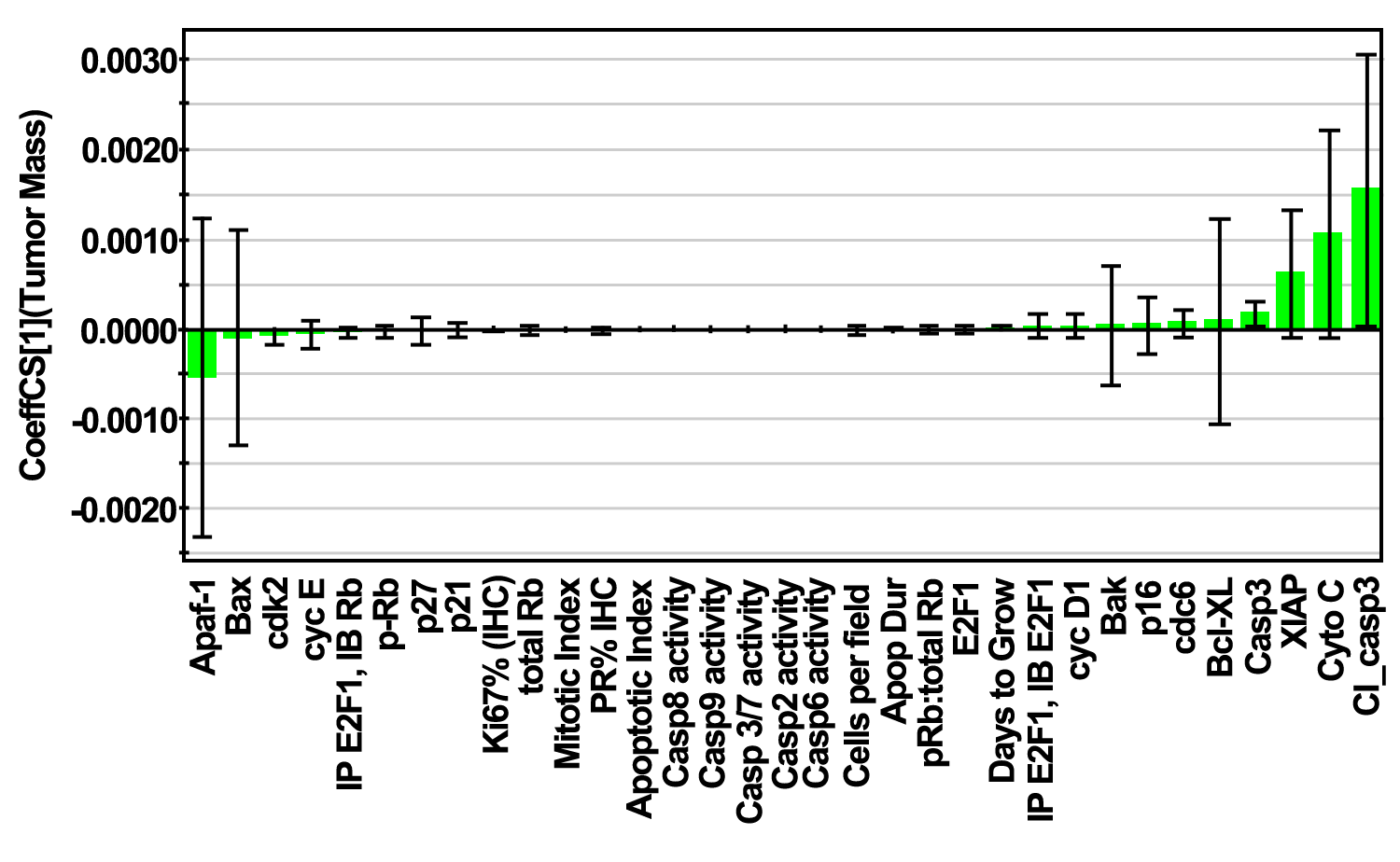


**Figure S2.** Results of PLS for Tumor Mass. Partial least squares projections to latest structures (PLS) analysis of DR and DS tumor growth characteristics. Coefficients for the interaction of DR X variables in the first component are shown for tumor mass. Coefficients are shown with jack-knifed 95% confidence intervals. Apoptotic markers display stronger correlation with tumor growth characteristics than do proliferative markers.
